# Cardiovascular health knowledge, attitude and practice among school-going adolescents and the availability of digital prerequisites for health education in Bhaktapur, Nepal

**DOI:** 10.1371/journal.pone.0323698

**Published:** 2025-06-25

**Authors:** Dayana Shakya, Nawi Ng, Natalia Oli, Abhinav Vaidya, Alexandra Krettek

**Affiliations:** 1 Department of Internal Medicine and Clinical Nutrition, Institute of Medicine, Sahlgrenska Academy at University of Gothenburg, Sweden; 2 Department of Nursing, Kathmandu Medical College, Nepal; 3 School of Public Health and Community Medicine, Institute of Medicine, Sahlgrenska Academy at University of Gothenburg, Sweden; 4 Department of Community Medicine, Kathmandu Medical College, Nepal; 5 Department of Public Health, School of Health Sciences, University of Skövde, Sweden; 6 Department of Community Medicine, Faculty of Health Sciences, UiT The Arctic University of Norway, Norway; Queensland University of Technology, AUSTRALIA

## Abstract

**Background:**

In Nepal, the proportion of annual deaths from cardiovascular disease (CVD) increased from 12% in 1990 to 22% in 2021. Although CVD manifests in adulthood, exposure to risk factors begins early in life. In Nepal, a high prevalence of risk factors combined with limited knowledge about cardiovascular health warrants a life course approach. One strategy could be a digitalized health education targeted at adolescents to prevent future CVDs.

**Methods:**

We conducted a cross-sectional survey to assess adolescents’ knowledge, attitude and practice (KAP) regarding cardiovascular health and explored possibilities for digital education. In total, 649 adolescents in grades 8–10 from two public and seven private schools in a semi-urban community of Nepal were surveyed. A self-administered questionnaire assessed KAP, digital prerequisites such as mobile phone use and internet availability at home, and gaming behaviors. Quantile regression was performed to assess the relationship among the variables.

**Results:**

The median scores were 69.1% (Interquartile range/IQR 63.1%–74.4%) for knowledge about cardiovascular health, 77.9% (73.3%–82.3%) for attitude and 76.7% (72.2%–81.1%) for practice. Quantile regression showed that the knowledge score for grade 9 adolescents was 4.2 percentage point (pp) higher (*p* < 0.001) and grade 10 adolescents was 4.0pp higher (*p* < 0.001) than those in grade 8. Knowledge was 4.9pp higher (*p* < 0.001) for private than for public school adolescents. Attitude scores were 2.0pp higher (*p* = 0.001) for girls than for boys and 1.7pp higher (*p* = 0.008) for private than for public school adolescents. For practice, boys scored 2.2pp higher (*p* < 0.001) than girls and private school adolescents scored 2.5pp higher (*p* < 0.001) than public. Furthermore, 98.6% of adolescents had smartphone access, 91.5% had internet access and 68.0% played mobile games.

**Conclusion:**

Adolescents have lower knowledge than attitude and practice regarding cardiovascular health. This combined with high digital access provides opportunities for digital health education, especially in public schools.

## Introduction

The prevalence of cardiovascular diseases (CVDs) is increasing in Nepal, and the proportion of annual deaths caused by CVD increased from 12.2% in 1990 to 22.0% in 2021 [1]. Cardiovascular risk factors like smoking, excessive alcohol use, insufficient intake of fruits and vegetables, obesity, high blood pressure, high blood sugar and high cholesterol levels are common in adults in Nepal; 98.2% of the population exhibit at least one of these risk factors [2].

Although CVDs manifest in adulthood, exposure to risk factors begins early in life. Cardiovascular risk factors, such as coronary intimal thickening, can manifest in foetal life [3–6]. Lifestyle patterns and habits connected to diet and physical activity are established in early childhood [7,8] and often persist into adulthood, which can contribute to cardiovascular events [9]. According to the most recent data available from the Global School-Based Student Health Survey Nepal 2015, 41% of adolescents aged 13–17 years eat fruit less than once a day and 32% eat vegetables less than once per day. In addition, 50% are physically inactive, 9% are current smokers, 11% are underweight and 7% are overweight or obese [10]. A recent study in eastern Nepal showed that 21% of adolescents aged 15–19 years had pre-hypertension and 7% were hypertensive [11]. Health promotion along with prevention strategies, such as screening and lifelong personalized management of risk factors and lifestyle interventions, are likely to positively impact cardiovascular disease incidence and its effects on personal, social and economic outcomes [7,8].

For this reason, the American Heart Association (AHA) published paediatric-focused guidelines for cardiovascular health promotion that emphasize the promotion of cardiovascular health throughout the life course. The guidelines define four favorable health behaviors related to smoking, body mass index, physical activity and healthy diet status and three favorable health factors related to blood pressure, total cholesterol and fasting blood glucose levels [12].

Despite the high prevalence of CVD and its risk factors, the knowledge, attitude and practice (KAP) regarding CVD among adolescents in Nepal is poor. Around 37% of adolescents in Kathmandu [13] and 49% in Hetauda [14] have good knowledge, which suggests that the majority of adolescents have insufficient understanding. Adolescents from metropolitan cities have better knowledge than those from rural municipalities [15]. Positive attitudes regarding CVDs have been reported for only one-to-two-thirds of adolescents [13,14]. Only 34–39% of adolescents in Nepal meet the recommended levels of physical activity [16]. Even children aged 5–10 years exhibit low physical activity and prefer unhealthier food items [17].

Targeting adolescents for improving cardiovascular health awareness may be accomplished through a medium that is user friendly and easily accessible to this population. Mobile-based health education could be one way to reach this technologically inclined group. To achieve this, the KAP gaps need to be identified in the target population to inform the development of digital health education. The AHA Guide for Improving Cardiovascular Health at the Community Level encourages the use of contemporary health education methods such as electronic gaming for school-aged children [18]. Before such education is implemented, it is also important to assess the digital prerequisites (smartphone availability and internet access), as well as the acceptability and usability of any proposed education in a low-and-middle-income country (LMIC) with limited resources like Nepal. Hence, the aim of this paper was to assess adolescents’ KAP regarding cardiovascular health and their access to prerequisites for digital cardiovascular health education in Nepal.

## Materials and methods

### Study design

We conducted a descriptive cross-sectional study among adolescents in grades 8–10 at two public and seven private schools in the Jhaukhel-Duwakot Health Demographic Surveillance System (JD-HDSS) in Nepal [19].

### Study setting

This study was conducted in the JD-HDSS, situated in the Changunarayan municipality of the Bhaktapur district, 13 km from Kathmandu, the capital of Nepal [19]. Research on various health-related issues has been carried out in this area previously, including smoking behaviour among adolescents [20], the KAP of the general population regarding CVD [21], physical activity among the general population [22] and the food preferences of children [17]. JD-HDSS was also the site for the Heart-Health Associated Research, Dissemination and Implementation in the Community (HARDIC) trial [23], which focused on educating mothers about the diets and physical activity of their young children. With an emphasis on a life course approach, the next step would be to focus on adolescents in the JD-HDSS.

Both public and private schools in the JD-HDSS we included in the study. School type was taken as a proxy for socioeconomic status [24].

### Study population, sampling and sample size

We obtained a list of public schools in the JD-HDSS from the District Education Office, Bhaktapur, and the list of private schools was obtained from the principal of one of the private schools. Public and private schools that provided education up to grade 10 were selected for inclusion. Both school types follow the same curriculum. Of the two public and eight private schools offering grade 8–10 classes in the study area, we included all but one private school.

There were 865 adolescents studying in grades 8–10 in the selected schools. Among them, 683 (79%) consented to participate. Eighteen were absent during data collection and there were 16 incomplete forms; 649 participants were included for analysis.

A power analysis was conducted for a one-sample binomial test to detect a difference in proportion of adolescents with good knowledge, attitude and practice from 0.60 (null hypothesis) to 0.70 (alternative hypothesis), with a sample size of 649. Using a two-sided test and a nominal significance level of 0.05, the estimated power was 0.9996, with an actual alpha of 0.0451. This indicates excellent power to detect the specified effect size.

### Data collection

A structured questionnaire was developed with references from the WHO STEPS survey [25] and the Global School-Based Student Health Survey 2015 [10]. The tool included questions about diet, physical activity, smoking and alcohol intake. It also included a section investigating facilitators for and barriers to eating a healthy diet and being physically active. We translated the tool into the Nepali language and used the Nepali version of the tool for data collection. Due to the limited number of schools, we pretested the questionnaires in one private school in JD-HDSS and one public school outside JD-HDSS.

After pretesting the tool, we made only minor changes that included simplifying words to make them understandable for the adolescents and adding pictures inside the tool instead of on separate showcards, which were not feasible for a self-administered questionnaire. The pretested schools were excluded from the data analyses. The final tool consisted of 13 knowledge questions, 26 attitude questions and 24 practice questions. A 9-item questionnaire examined whether the adolescents had the necessary prerequisites for digital education, such as smartphones and internet access.

After the schools were selected, multiple visits, phone calls and meetings with the principals of each school were conducted. We received written permission from the school authorities to conduct the study. Data were collected from June 6 to November 25, 2022.

A research assistant with a master’s of science degree in nursing and four enumerators with a bachelor’s of science in nursing were trained for data collection by the first author, including paper-based questionnaires and anthropometric measurements. The first author guided and supervised the research assistant and enumerators in all fieldwork while the research assistant was responsible for planning the logistics of the fieldwork. The enumerators met the adolescents in their classes and distributed the questionnaires. Adolescents were given 30 minutes to complete the questionnaire before it was collected. The enumerators then measured the height, weight and hip and waist circumference of the adolescents using the WHO-recommended techniques [26]. Briefly, height was measured using a stadiometer (plastic stadiometer HMS PL). Weight was measured using a Rossmax digital weighing machine. Hip circumference was taken with a non-elastic measuring tape at the widest part of the buttocks with feet together. Waist circumference was also measured using a non-elastic measuring tape at the middle point between the bottom of the last rib and the top of the hip bone.

### Ethical considerations

The study was approved by the Nepal Health Research Council (Registration number 660/2021). Written parental consent and the children’s assent were obtained before they participated in the study. We sent the consent form and the information sheet in the Nepali language home with the adolescents for their parents to sign and collected it the next day. Among the 865 adolescents, 683 (79%) received parental consent. The adolescents were allowed to withdraw from the study at any time without any explanation. Confidentiality was maintained by coding the questionnaires and separating the identifiers from the student data. The identifiers were saved separately on a hard disk kept in a locked cabinet in the principal investigator’s office.

### Statistical analysis

After verifying the completeness and out-of-range scores, the data were entered, organized and coded using the Statistical Package for Social Sciences (IBM SPSS Statistics version 28). For multiple-choice questions, four options were given, with one correct answer. For multiple-response questions, multiple correct and incorrect options were given, and respondents had to tick the responses that they thought were correct. For knowledge, each correct answer was scored 1 and each incorrect answer was scored 0; there was no negative scoring. For attitude, a five-point Likert scale was used, with a scale of strongly agree (5) to strongly disagree (1). Reverse coding was conducted for negatively framed questions.

We followed the AHA guidelines for scoring practice [18,27,28]. These included questions regarding daily consumption of fruits and vegetables, daily physical activity, and smoking and drinking behaviors. We asked the adolescents to tick how many days they had consumed fruits, vegetables, carbonated soft drinks, and food high in salt, sugar and fats in the previous week. We then asked them how many servings they had consumed in each category on those days. For physical activity, we asked them how many minutes they were physically active per day on average, including both home and school time. For tobacco and alcohol use, we asked them if they had ever used tobacco or alcohol, how many days they had used these products in the past seven days and how much of these products they had used on those days.

We added individual scores for each domain to obtain ‘raw’ scores for diet, physical activity and tobacco/alcohol use. The domain scores were converted into a scale of 0–100 for each domain. These domain scores were then added and converted into percentages to obtain the final percent scores for knowledge, attitudes and practices. Normality testing using the Shapiro–Wilk test was significant (p < 0.001), suggesting that the data were not normally distributed. Therefore, descriptive statistics such as frequency, percentage, median and inter-quartile range were calculated. The chi square test and Mann–Whitney U test were used to find differences between the school types. Multivariable quantile regression was used to assess the association of KAP with independent variables like sex (boys/girls), school type (public/private) and grade (8/9/10). The significance level was determined at 5% (p < 0.05). In this study, data were collected on age, school class, and school type. As only these three independent variables were available, they were included in the regression model. The effect of sex on the outcome variable was estimated within the model; therefore, no subgroup analysis was conducted.

## Results

### Characteristics of the study participants

The characteristics of the participants are presented in Table 1. The mean age of the adolescents was 14.5 ± 1.1 years. Half of the adolescents were boys, and 60% were from private schools. The sample was distributed almost equally among the grades.

**Table 1:** Characteristics of study participants.

Characteristics	Public school n = 262 n (%)	Private school n = 387 n (%)	Total n = 649 n (%)
**Age (years)** <13 13–15 >15	4 (1.5) 180 (68.7) 78 (29.8)	10 (2.6) 337 (87.1) 40 (10.3)	14 (2.2) 517 (79.7) 118 (18.2)
**Sex** Boys Girls	116 (44.3) 146 (55.7)	215 (55.6) 172 (44.4)	331 (51.0) 318 (49.0)
**Grade** Eight Nine Ten	68 (26.0) 100 (38.2) 94 (35.9)	137 (35.4) 134 (34.6) 116 (30.0)	205 (31.6) 234 (36.1) 210 (32.4)

The anthropometric measurements of the boys and girls in public and private schools are shown in a supplementary table (S1 Table). The age-standardized z scores for BMI show that there were no significant differences neither according to sex nor school type.

### Cardiovascular health knowledge, attitudes and practices

The median score for the adolescents’ knowledge of cardiovascular disease was 69.1% (63.1–74.4), the median attitude score was 77.9% (73.3–82.3), and the median practice score was 76.7% (72.2–81.1). Most of the adolescents were aware that fruits (90.1%) and vegetables (88.4%) are healthy for the heart. Fast food (sausages and burgers) was considered unhealthy by 98.3% and instant noodles by 99.1%. Although 80.9% of the adolescents identified red meat as unhealthy, only 22.5% recognized that fish is a heart-healthy food.

Less than half of the adolescents correctly identified the risk factors for CVD, including high blood pressure (47.6%), lack of exercise (41.6%), obesity (46.5%), hereditary predisposition (46.1%) and stress (50.1%). Less than one-third recognized that high blood sugar (30.5%), high blood lipids (30.5%) and sedentary lifestyle (28.5%) can lead to CVD. Only 20% of the adolescents were aware of the healthy plate concept [29], and only 4% were aware of the AHA recommendation to eat five servings of fruits and vegetables per day.

Only 55.2% of the adolescents were aware of the recommended physical activity levels for their age group. Even fewer knew that physical activity increases the strength of the heart (50.8%) and helps burn fat (48.2%). Around half of the respondents (45.5%) could not identify sedentary behaviour from the given list of activities and were not mindful that they needed to engage in physical activity after every hour of sitting.

Few adolescents identified that smoking can lead to hypertension (29.9%) and diabetes (8.2%). Around half could identify tobacco use (52.5%) and alcohol consumption (58.7%) as risk factors for developing CVD. Most of the adolescents recognized that smoking is harmful (98.6%) and can cause cancer (95.7%) and lung diseases (81.5%).

Only 12.3% of adolescents ate the AHA-recommended five servings of fruits and vegetables per day. On average, they consumed 0.9 servings of fruit and 2.0 servings of vegetables daily. They were physically active for an average of 69 minutes (IQR: 36–180 mins) per day. In private schools, the adolescents scored significantly better in the categories of consumption of food with a high salt, fat or sugar content, physical activity and using smokeless tobacco (Table 2).

**Table 2:** Practices for preventing cardiovascular disease among adolescents by school type.

Activity	Public school n = 262 n (%)	Private school n = 387 n (%)	*^p*-value
Eat ≥ 5 servings of fruits and vegetables per day	26 (9.9)	54 (14.0)	0.125
Drink soft drinks <1 time per day	171 (65.3)	245 (63.3)	0.610
Eat food with high salt content like chips, crackers, *daalmoth*, *paapad* <1 time per day	74 (28.2)	138 (35.7)	**0.048**
Eat food with high fat content like ghee, fried food <1 time per day	103 (39.3)	198 (51.2)	**0.003**
Eat food with high sugar content like chocolate, *mithai* <1 time per day	75 (28.6)	156 (40.3)	**0.002**
Physically active for ≥60 mins per day	142 (54.2)	263 (68.0)	**<0.001**
Never smoked	252 (96.2)	371 (95.9)	0.840
Never used smokeless tobacco	261 (99.6)	378 (97.7)	**0.049**
Never used any alcohol-containing drinks	211 (80.5)	319 (82.4)	0.541

*Daalmoth:* a traditional crispy, spicy, salty dry snack made from deep fried lentils, nuts, spices and oil.

*Paapad:* a thin, crispy snack made from dried lentils.

*Mithai:* traditional Nepali sweets like *jeri*, *barfi.*

^Chi square test.

We found that the median knowledge scores were 73.9% (69.6–82.6) for diet, 77.7% (66.7–83.3) for physical activity and 70.9% (61.3–77.4) for alcohol/tobacco use. The median attitude scores were 75.5% (71.1–82.2) for diet, 76.6% (70–83.3) for physical activity and 80.0% (74.5–85.5) for alcohol/tobacco use. The median practice scores were 66.7% (56.7–76.7) for diet, 60.0% (60–70) for physical activity and 100% (100–100) for alcohol/tobacco use. The median scores for knowledge, attitudes and practices regarding cardiovascular health by school type and CVD risk factors are shown in Table 3.

**Table 3:** Categorization of KAP on cardiovascular health among adolescents by risk factors and school type (n = 649).

	Public school n = 262 Median	Private school n = 387 Median	*^p*-value
**Knowledge median score (%)**			
Overall	66.9	70.8	**<0.001**
Diet	73.9	78.3	**<0.001**
Physical activity	72.2	77.7	**<0.001**
Alcohol/tobacco use	70.9	70.9	0.223
**Attitude median score (%)**			
Overall	77.4	78.5	0.113
Diet	75.5	77.7	0.182
Physical activity	76.6	76.6	0.676
Alcohol/tobacco use	80.0	81.8	**0.021**
**Practice median score (%)**			
Overall	75.5	77.7	**<0.001**
Diet	63.3	70.0	**<0.001**
Physical activity	60.0	70.0	**<0.001**
Alcohol/tobacco use	100	100	0.847

^Mann–Whitney U test.

Multivariable quantile regression analysis showed that although knowledge scores did not differ by sex, adolescents in grade 9 scored 4.2pp higher than those in grade 8, and students in grade 10 scored 4.0pp higher than those in grade 8 when adjusted for sex and school type. Knowledge scores were 4.9pp higher among private school adolescents when adjusted for sex and grade. Attitude scores were 2.0pp higher among girls when adjusted for grade and school type and 1.7pp higher among adolescents in private schools when adjusted for sex and grade. Although there was no difference in practice scores according to grade, girls scored 2.2pp lower than boys when adjusted for grade and school type. Adolescents at private schools scored 2.2pp higher in practice than public school adolescents when adjusted for sex and grade (Table 4).

**Table 4:** Multivariable quantile regression analysis for association of knowledge, attitude and practice percent score regarding cardiovascular health with independent variables.

Variables	Knowledge			Attitude			Practice		
	Coefficient	p-value	95% CI	Coefficient	p-value	95% CI	Coefficient	p-value	95% CI
**Sex** **Girls**	0.53	0.538	−1.2–2.2	2.0	**0.001**	0.8–3.2	−2.2	**<0.001**	−3.2 – −1.2
**Grade** **9** **10**	4.2 4.0	**<0.001** **<0.001**	2.2–6.3 1.9–6.1	−0.3 0.2	0.69 0.79	−1.8–1.2 −1.3–1.7	<−0.001 <−0.001	1.0 1.0	−1.2–1.2 −1.2–1.2
**School type** **Private**	4.9	**<0.001**	3.2–6.6	1.7	**0.008**	0.4–2.9	2.2	**<0.001**	1.2–3.2

Adolescents thought that they would be motivated to eat healthier foods if they had access to better information about such food (59.0%) and if family members were eating healthy food (53.0%). However, 50.4% of adolescents said that they usually ate whatever was given to them and 34.0% of adolescents perceived that there were no barriers for eating healthy.

Adolescents mentioned that better information about the importance of physical activity (61.0%) and support from family and friends (42.5%) would motivate them to be active. They identified that a lack of leisure time (50.8%) and a lack of parks and gyms (40.1%) hindered their physical activity. The facilitators for and barriers to healthy diet and physical activity are shown in a supplementary table (S2 Table).

### Availability of prerequisites for digital education

The adolescents were asked if they had access to smartphones and the internet. While 98.6% of the adolescents had access to smartphones, only half had their own. Among those with smartphones available in the family, 76% of the adolescents had access to an Android phone. Ninety-two percent had access to the internet at home. More than two-thirds of the adolescents (68.0%) reported playing mobile-based games, with 22.7% playing mobile games daily.

Although more public-school adolescents owned a smartphone, private school adolescents had more access to smartphones, either through having their own or through family members. Additionally, more private school adolescents had internet access than public school adolescents (Table 5).

**Table 5:** Digital prerequisites for cardiovascular health education by school type (n = 649).

	Public school n (%)	Private school n (%)	*p*-value*^*
Availability of smartphones in the family			
Have their own smartphone	150 (57.3)	177 (45.7)	0.002*
Family members have smartphones	106 (40.5)	207 (53.5)	
No smartphone in the family	6 (2.3)	3 (0.8)	
Type of smartphone among those who have access (n = 640)			
No smartphone	6 (2.3)	3 (0.8)	<0.001
Use Android-based smartphone	216 (82.4)	279 (72.1)	
Use iPhone	23 (8.8)	47 (12.1)	
Use both Android phone and iPhone	17 (6.5)	58 (15.0)	
Have access to internet	221 (84.4)	373 (96.4)	<0.001
Permission to use smartphone			
Allowed to use smartphone without restriction	56 (21.4)	85 (22.0)	0.089
Allowed to use smartphone with restriction	151 (57.6)	225 (58.1)	
Allowed to use smartphone only on weekends	39 (14.9)	68 (17.6)	
Never allowed to use smartphone	16 (6.1)	9 (2.3)	
Play mobile-based games	166 (63.4)	272 (70.3)	0.065

^Chi square test*Fischer exact test.

## Discussion

We found that there was lower overall knowledge about cardiovascular health among adolescents in comparison to attitude and practice in a semi-urban community of Nepal. Additionally, practice regarding diet and physical activity were lower compared to smoking and alcohol intake. The lower knowledge but higher attitude and practice can be explained by the self-perception theory [30] which suggests that people develop their attitudes, emotions, and beliefs by observing their own behavior and then making inferences about what they must be feeling or thinking. This finding in our study is also supported by social cognitive theory [31] which emphasizes the role of observational learning, imitation, and modeling in behavior development suggesting that adolescents’ practice may result from imitating others despite their own low knowledge.

The low level of knowledge regarding cardiovascular health among adolescents may be attributed to a lack of regular school-based awareness campaigns or health intervention programmes [15], a lack of health literacy [32] and a low perceived relevance of CVD for adolescents, as explained by the health belief model [33]. The low knowledge of the healthy plate concept and the five servings of fruits and vegetables among the adolescents corresponds with their overall low knowledge of nutrition [34].

The overall positive attitude regarding CVDs in our study aligns with earlier studies inside and outside Nepal [13–15,34]. In one study in China, 82.1% of adolescents expressed a willingness to adopt a healthy lifestyle [35]. Cultural and societal influences and an individual’s education may influence their attitudes. Good attitude levels mean that adolescents understand the harm of CVDs and are positive about preventive strategies.

In our study, the KAP scores showed that healthy diet practices were lower than the adolescents’ knowledge of these practices. Only 12.3% of adolescents in our study ate the AHA-recommended five servings of fruits and vegetables per day. Although this is better than reports in the Nepali national data on adolescents [10], low consumption patterns in adults (3.3%) are reflected in their children [25]. Global trends show similar findings [12], with low consumption of fruits and vegetables among adolescents, especially in South and East Asia [36]. Despite knowledge about its harmful consequences, consumption of junk food and soft drinks is high among adolescents in Nepal. This could be due to easy availability [37,38], accessibility through ready-to-use packaging [38], addictive tastes [37], propaganda advertising [37], and a lack of specific policy regulations on junk food [39]. In our study, the adolescents described several barriers to healthy eating: having to eat what is offered, not wanting to give up their favorite foods and the food preferences of adult family members.

Although our study demonstrated that 62.4% of adolescents were physically active for the recommended 60 minutes or more per day, the national data from Nepal show that only 15.1% are physically active [10]. One reason for this discrepancy may be that, especially private schools in our study offered physical activity in the form of gymnastics and dancing, karate or taekwondo classes, and team sports like basketball, football and volleyball. This may explain the difference in observed physical activity between the two school types and also a higher physical activity than the national average. In addition, the different data collection tools used in the two studies may have contributed to the differences in the findings. While the national data focus on moderate-to-vigorous-intensity physical activity for 60 minutes per day, the adolescents in our study may have also included low-intensity physical activity in their answers.

Similar to Nepal, global physical activity trends are declining [40], especially since COVID-19 [41]. Evidence-based solutions recommend promoting physical activity through school support, social and digital environments, and multi-utility urban environments to urgently improve physical activity among adolescents [42]. Despite the higher physical activity reported in our study, adolescents mentioned the lack of leisure time, parks and gyms as barriers to being physically active.

In line with increasing global trends in smoking and alcohol consumption among adolescents, the national Nepalese data show that 5.0% are current smokers and 4.6% are current alcohol users [10]. Our study revealed similar data. The increasing trends in smoking stem from increased susceptibility to smoking among adolescents, mainly due to family and environmental factors [20], as well as tobacco advertising, promotion and sponsorship [43]. We found that the knowledge scores for tobacco use and alcohol consumption were lower than the corresponding practice. The lower levels of tobacco and alcohol consumption may be due to underreporting caused by social stigma and cultural norms [44,45].

In our study, the adolescents in private schools had better knowledge, attitudes and practices than those in public schools. School type can be taken as an indicator of socioeconomic status in Nepal; parents with higher socioeconomic status and better education usually enroll their children in private schools [24]. Private schools often have better resources and more qualified teachers [46,47]. In the JD-HDSS, public schools do not charge monthly tuition fees, while private schools charge between NPR 2,500 and NPR 4,500 per month (USD 18–33). Based on the government-set minimum monthly wage of NPR 17,300 (USD 127) as of July 2023 [48], private school tuition represents approximately 14–26% of a worker’s monthly income. For context, Nepal’s per capita GDP for fiscal year 2022–2023 was USD 1378 [49], or about USD 115 per month. Socioeconomic status can influence health awareness, and individuals from higher socioeconomic backgrounds often have better access to health information and resources [50]. Compared to adolescents in grade eight, those in grades nine and ten had better knowledge about CVD, likely due to higher maturity, cumulative learning over the years and increased access to information.

Our study also showed that girls had better attitudes than boys with regard to CVDs. This can be explained by social role theory, which posits that societal expectations and roles assigned to genders significantly influence behaviors and attitudes [51]. Moreover, in the Nepalese context, smoking and alcohol consumption are considered acceptable for men but not for women. This could have shaped the adolescents’ attitudes, and hence, there may have been an underreporting of smoking and alcohol consumption [44]. Our study also showed that boys had better practices regarding cardiovascular health than girls. This may be due to the higher physical activity practices among boys [52].

Besides, sex, grades and school type, other factors such as parents’ education [53] and occupation [54], monthly family income [54], parental risk behaviors [55], and history of chronic illness in the family [56,57] could also affect the KAP of the adolescents.

Given the life course approach for the prevention of CVDs, and to meet the Sustainable Development Goal 3.4, immediate measures targeted at adolescents are necessary. These include integrating health education about noncommunicable diseases in school curricula, establishing school health clubs, organizing health campaigns and improving access to healthy foods and physical activity in schools, as also emphasized in the Multisectoral Action Plan for Prevention and Control of Noncommunicable Diseases II (MSAP II) [58].

Multiple school-based programs have been successfully implemented. School health and nutrition program (SHNP) [59] by the government of Nepal has provided midday meals that has not only improved the nutritional status of the children but also the school attendance. The ‘Health promoting schools initiative’ within the SNHP creates supportive environments for students to learn and grow by integrating health promotion into the school setting such as incorporating health education into the school curriculum, promoting physical activity through sports and exercise, improves nutrition standards in school meals and creates a smoke-free, substance-free environment. Other programs such as Adolescent Sexual and Reproductive Health (ASRH) Program [60]; Water, Sanitation, and Hygiene (WASH) in Schools [61] program; and Mental health awareness campaigns [62] endorsed by the government of Nepal have proved to be fruitful. However, a more focused approach in these campaigns is necessary targeting CVD and NCDs to raise awareness in this sector.

The effective implementation of any digital health programme in LMICs requires an assessment of the necessary digital infrastructure–that is, stable electricity, affordable mobile and internet services, and a good understanding of the socioeconomic context [63,64]. In our study, half of the adolescents had their own smartphones and 48% had access to smartphones through their family members. The literature on mHealth in LMICs provides evidence of the ubiquity of mobile phones and ever-increasing connectivity, even reaching remote populations [65].

Our study showed that the majority of the adolescents (76%) had access to Android-based phones. This is comparable to the data from the monthly market share of mobile operating systems in Nepal from March 2021 to March 2024, which shows an 85.6% Android predominance [66]. Future digital education efforts therefore need to account for this predominance. Furthermore, 92% of adolescents had access to the internet at home. According to the Nepal Telecommunications Authority, Nepal has reached a broadband penetration rate of 144.56% [67] with many using internet services from more than one service provider. This increasing connectivity, coupled with increasing mobile access, provides numerous opportunities for mobile-based digital health interventions in Nepal.

However, digital connectivity in terms of smartphone and internet access differed significantly between the school types. This should be taken into account when digital school-based interventions are planned.

## Strengths and limitations

A strength of this study is that it not only assessed the KAP on cardiovascular health among adolescents but also evaluated the digital prerequisites for digital health education. Furthermore, our study included a comparison of public and private schools.

The study was conducted in JD-HDSS, a semi-urban to urban area outside Kathmandu. One limitation is that the results may not be generalizable to other settings. In addition, the cross-sectional nature of the study can limit the ability to draw causal inferences between the variables. We ensured that no teacher was present during data collection and assured the adolescents that the data collected would be confidential and would not affect their grades. Although these measures were taken to minimize errors and increase response rates, the self-administered nature of data collection may contribute to reporting bias. Taking school type as a socioeconomic proxy may not adequately represent the socioeconomic status of the adolescents. Although the regression model included age, school class, and school type, the possibility of residual confounding cannot be excluded due to the absence of other relevant covariates, such as socioeconomic status, parental education or occupation, or access to health information.

## Conclusion

Our study showed that adolescents had lower knowledge than attitude and practice regarding cardiovascular health. Adolescents studying in private schools had better knowledge, attitude and practice, signifying the need for better health education in public schools. Increasing mobile usage and internet access in an LMIC like Nepal provides numerous opportunities for digitalized health education targeted at adolescents, especially through school-based interventions in both public and private schools.

## Supporting information

Table S1 Anthropometric measurements of adolescents in grades 8–10. (DOCX)

Table S2 Facilitators for and barriers to healthy diet and physical activity among adolescents in grades 8–10. (DOCX)

S3 Inclusivity in global research questionnaire. (DOCX)
