# Supplementary material for: Cardiovascular health knowledge, attitude and practice among school-going adolescents and the availability of digital prerequisites for health education in Bhaktapur, Nepal
Source: PLoS One. 2025 Jun 25;20(6):e0323698. doi: 10.1371/journal.pone.0323698 (PMC12193904; doi:10.1371/journal.pone.0323698)
Supplement: Table S1 — (DOCX) [file pone.0323698.s001.docx]

**S1 Table: Anthropometric measurements of adolescents of grades 8-10** (**n=649)**

| **Characteristics** | **Public school** | | **Private school** | | **Total** | |
| --- | --- | --- | --- | --- | --- | --- |
|  | **Boys** | **Girls** | **Boys** | **Girls** | **Boys** | **Girls** |
| **Mean height (cm)** | 161.2±7.8 | 152.3±5.6 | 161.5±8.4 | 152.9±5.8 | 161±8.2 | 152±5.7 |
| **Mean weight (kg)** | 49.6±10.2 | 47.1±8.1 | 49.3±11 | 46±8.1 | 49±10.7 | 46.5±8.1 |
| **Mean waist circumference (cm)** | 66.2±7.1 | 65.7±6.7 | 67.9±8.7 | 64.4±7.1 | 67±8.2 | 65±6.9 |
| **Mean hip circumference (cm)** | 82.3±7.2 | 85.9±6.8 | 83.9±8.2 | 85.8±6.9 | 83±7.9 | 86±6.8 |
| **Mean WHR** | 0.8±0.1 | 0.76±0.1 | 0.8±0.1 | 0.75±0.1 | 0.8±0.1 | 0.76±0.1 |
| **Mean BMI** | 19±3.1 | 20.3±3.1 | 18.7±3.3 | 19.6±3.2 | 18.8±3.2 | 19.9±3.2 |
| **BMI**  Underweight (<18.5)  Normal (18.5-25)  Overweight (>25) | **n (%)**  59 (51)  53 (45.7)  4 (3.4) | **n (%)**  41 (28.1)  94 (64.4)  11 (7.5) | **n (%)**  123 (57.2)  79 (36.7)  13 (6.1) | **n (%)**  68 (39.5)  95 (55.2)  9 (5.3) | **n (%)**  182 (55)  132 (40)  17 (5.1) | **n (%)**  109 (34.3)  189 (59.4)  20 (6.3) |

WHR: Waist hip ratio, BMI: Body mass index
