# Supplementary material for: Cardiovascular health knowledge, attitude and practice among school-going adolescents and the availability of digital prerequisites for health education in Bhaktapur, Nepal
Source: PLoS One. 2025 Jun 25;20(6):e0323698. doi: 10.1371/journal.pone.0323698 (PMC12193904; doi:10.1371/journal.pone.0323698)
Supplement: Table S2 — (DOCX) [file pone.0323698.s002.docx]

**S2 Table: Facilitators for and barriers to healthy diet and physical activity among adolescents in grades 8–10** (**n=649)**

|  | **Facilitators** | **n (%)** | **Barriers** | **n (%)** |
| --- | --- | --- | --- | --- |
| **Diet** | Better information about food/healthy eating  Family members eating healthier food  Own ill health  Advice from a doctor or nurse  Better shops in the local area  Disease or death of a close relative  More money as healthier food cost more  Don’t know  Nothing | 385 (59.3)  343 (52.9)  243 (37.4)  239 (36.8)  113 (17.4)  57 (8.8)  45 (6.9)  23 (3.5)  21 (3.2) | I eat what is given to me in the family  No difficulty trying to eat healthier  I don’t want to give up foods that I like  Taste preferences of adults in the house  Don’t know  I am not motivated enough to eat healthy  Don’t know enough about healthy eating  Healthy foods are costly | 327 (50.4)  221 (34.1)  134 (20.6)  90 (13.9)  87 (13.4)  48 (7.4)  43 (6.6)  35 (5.4) |
| **Physical activity** | Better information about importance of physical activity  Support by friends, relatives  Availability of parks, playgrounds, gym  Own ill health  Advice from a doctor or nurse  Disease or death of a close relative  Don’t know  Nothing | 396 (61)  276 (42.5)  237 (36.5)  188 (29)  170 (26.2)  45 (6.9)  32 (4.9)  20 (3.1) | Lack of leisure time  Lack of parks or places for physical activity  Having other things to do  Laziness  Embarrassment in front of others  Don’t know  Caring for smaller siblings or elderly  Do not feel the need to do more physical activity  Own ill health | 330 (50.8)  260 (40.1)  236 (36.4)  187 (28.8)  124 (19.1)  71 (10.9)  71 (10.9)  53 (8.2)  58 (8.9) |
